# Complete genome sequence of *Limosilactobacillus reuteri* LU150, a potential vitamin B_12_ producer from the NORDBIOTIC collection

**DOI:** 10.1128/mra.00800-24

**Published:** 2024-11-21

**Authors:** A.K. Szczepankowska, B. Cukrowska, T. Aleksandrzak-Piekarczyk

**Affiliations:** 1 Institute of Biochemistry and Biophysics, Polish Academy of Sciences, Warsaw, Poland; 2 Department of Pathomorphology, The Children Memorial Health Institute, Warsaw, Poland; Loyola University Chicago, Chicago, Illinois, USA

**Keywords:** probiotics, *Limosilactobacillus reuteri*, obesity, hypercholesterolemia, vitamin B, propionic acid, bile salt hydrolase, cobalamin biosynthesis, fecal isolate, lactic acid bacteria

## Abstract

We performed whole-genome sequencing of *Limosilactobacillus reuteri* LU150, a human fecal isolate from the NORDBIOTIC collection. The genome consists of 2,039,406 bp with a GC content of 38.9%. The genetic content of LU150 suggests its probiotic potential.

## ANNOUNCEMENT

*Limosilactobacillus reuteri* is a gram(+) lactic acid bacterium commonly found in the gastrointestinal tract of humans and animals (1). *Limosilactobacillus reuteri* LU150, from Nordic Biotic Ltd., was originally isolated from a fecal sample of a healthy individual and is deposited in the DSMZ Collection (DSM33841).

Genomic DNA was isolated from LU150, which was grown overnight in MRS liquid medium (Oxoid) at 37°C under aerobic conditions, using a cetyltrimethylammonium bromide/lysozyme extraction method (2), with modifications (3). Whole-genome sequencing was performed using a hybrid approach, integrating Illumina short-read sequencing on the MiSeq platform (Illumina) with the NEB Ultra II FS library kit (New England Biolabs) in paired-end reads and Nanopore long-read sequencing on a GridION sequencer (Oxford Nanopore Technologies) with the SQK-LSK109 native barcoding expansion kit (EXP-NBD103) and R9.4.1 flow cell.

Illumina sequencing yielded 768,712 reads and 410,891,695 bases. After quality control (FASTQC v.0.12.0) (4) and filtering with trimming (fastp v.0.23.2) (5), 749,676 reads totaling 362,901,410 nt were retained. Nanopore sequencing produced 25,755 reads and 236,839,792 nt, which were based-called using Guppy v.6.1.3 in super accuracy mode, filtered (NanoFilt v.2.8.0) (6) to remove reads <1 kb and Q-score of <12, and had adapters removed by Porechop (v.0.2.4) (https://github.com/rrwick/Porechop). Quality checking with NanoPlot (v.1.41.6) (6) resulted in 22,584 reads totaling 235,246,629 nt, with an average read length of 10,416.5 bp and N_50_ of 15,297 bp.

Long-reads were assembled, circularized, and rotated using the Trycycler pipeline (v.0.5.3) (7) and polished using Medaka (v.1.7.2) (8). The assembly was further aligned with Illumina data and polished using Polypolish (v.0.5.0) (9) and POLCA (v.4.0.5) (10). Default parameters were used for all software executions. The final genome coverage was 291.0×.

The *de novo* assembly revealed a single circular chromosome of 2,039,406 bp with a GC content of 38.9%. The NCBI Prokaryotic Genome Annotation Pipeline (v.6.6) (11) identified 2,090 genes, including 2,000 coding sequences and 90 RNA genes. To infer cellular functions from protein sequences and match them with known biosynthetic pathways, BlastKOALA and KEGG Mapper tools were used (12, 13). We identified proteins for vitamin B synthesis pathways, including those for cobalamin (vitamin B_12_), thiamine (vitamin B_1_), and riboflavin (vitamin B_2_). The identification of a complete cobalamin biosynthesis pathway in LU150 is particularly noteworthy, as this feature is rare among lactic acid bacteria (14). Additionally, a partial pathway for propionic acid biosynthesis, converting 1,2-propanediol to propionic acid, was found. The LU150 strain also carries the *bsh* gene encoding bile salt hydrolase (BSH). It is known that bacterial BSH activity significantly impacts the systemic metabolic processes and adiposity in the host and represents a key mechanistic target for the control of obesity and hypercholesterolemia (15, 16).

## Data Availability

Complete sequencing data has been deposited in GenBank under BioProject: PRJNA1071652; BioSample: SAMN39639085. The whole genome sequence is available under accession number CP147911. lllumina SRA reads are available under accession number SRX23536625 and Oxford Nanopore SRA reads under SRX23536624.
